# A case report of congenital myasthenic syndrome caused by a mutation in theCHRNE genein the Iranian population

**Published:** 2020

**Authors:** Zahra FARJAMI, Negar KHODAENIA, Neshat EBRAHIMI, Gholamreza ZAMANI, Amir Hosein ASHNAEI, Mohammad GALEHDARI, Mehdi MORADYAR, Massoud HOUSHMAND

**Affiliations:** 1 National Institute of Genetic Engineering and Biotechnology, Tehran, Iran; 2 Laboratory of Cedars-Sinai Medical Center, Los Angeles, California, USA; 3 Pediatric Neurologist, Tehran University of Medical Sciences, Tehran, Iran; 4 Department of Biotechnology, Science and Research Branch, Islamic Azad University, Tehran, Iran; 5 Department of Modern Sciences and technologies; Faculty of Medicine, Mashhad University of Medical Sciences, Mashhad, Iran; 6 Department of Biology, Faculty of Sciences, North Tehran Branch, Islamic Azad University, Tehran, Iran

**Keywords:** Congenital myasthenic syndrome, CMS, CHRNE

## Abstract

Congenital myasthenic syndrome (CMS) refers to a heterogeneous group of inherited disorders, characterized by defective transmissionat the neuromuscular junction (NMJ). Patients with CMS showed similar muscle weakness, while other clinical manifestations are mostly dependent on genetic factors. This disease,caused bydifferent DNA mutations, is genetically inherited. It is also associated with mutations of genes at NMJ, involving the acetylcholine receptor (AChR) subunits. Here, we present the case ofa five-year-old Iranian boywith CMS, undergoingtargeted sequencing of a panel of genes, associated with arthrogryposis and CMS. The patient had six affected relatives in his genetic pedigreechart. The investigations indicated a homozygous single base pair deletion at exon 12 of the *CHRNE *gene (chr17:4802186delC).This region was conserved across mammalian evolution and was not submitted to the 1000 Genomes Project database.Overall, the *CHRNE*variant may beclassified as a significant variant in the etiology of CMS.It can besuggested thatthe Iranian CMS population carry regional pathogenic mutations, which can be detected viatargeted and whole genome sequencing.

## Introduction

Congenital myasthenic syndrome (CMS)refers to a heterogeneous group of inherited genetic disorders,affecting neuromuscular transmission. Although most symptoms of CMS appear within several days after birth or in early childhood,in rarecases,its onset may be delayed until childhood(1). CMSaffects not only the muscles movingthe eyes,butalso chewing and swallowingmuscles,involved in abnormal physical exhaustion(2).CMS is also commonly associated with dysfunctionalacetylcholine receptor (AChR)ε-subunits. Evidence shows that *CHRNE*gene mutations lead to AChRdeficiency (3). The encoded AChRprotein can be found in the cellular membrane of skeletal musclesat the neuromuscular junction (NMJ)(4). The sites, products, and disorders of CMS-related genes are presented in Table 1. 

The severity of CMS is highly variable, ranging from minor symptoms to progressive disablingweakness. Although the prevalence of this syndrome is unknown, it has beenclassified asa rare disease (6).The diagnosis of CMS is established,based on clinicalfindings, electromyography, genetic tests, andmeasurement of serum AChRantibodies. All treatment protocolsinvolve acetylcholinesteraseinhibitors(9). Targeted sequencing represents a cost-effective approach to detect the variants inmultiple or large genes.

In the present case report, we aimed to examine nucleotide variations in a panel of genes in anIranian male CMS patient.

## Case Presentation

**Figure 1:**
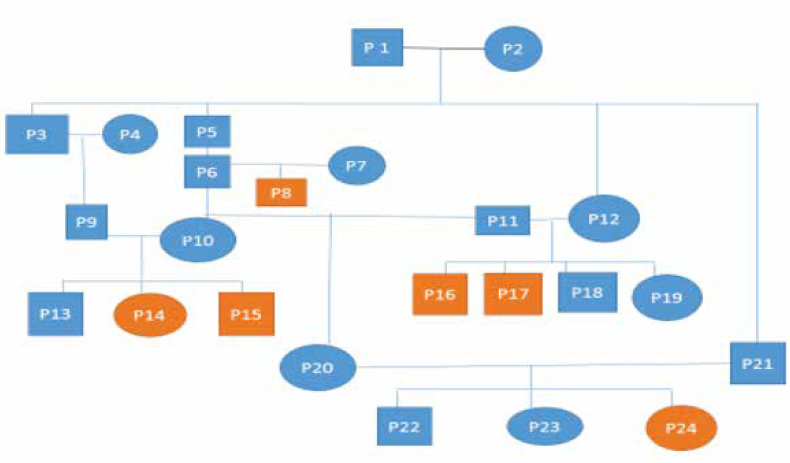
*The patient’s pedigreechart*

The patient was afive-year-old boy withat least sixCMS-affected relatives inhisfamily pedigree (Figure 1). His parents were second cousins. According to the neurology report,he was diagnosed withptosis at birth with diurnal variation and a high-arched palate. Themuscle biopsy with hematoxylin and eosin (H&E) staining revealed a striated muscle tissue with significant variations in fiber size. The atrophic fibers were roundedand/or angular and dispersed. The ATPase reaction at pH of 9.4, 4.6, and 4.35 revealed prominent atrophy of type II fibers.Also, measurement of blood cells showed a higher white cell count (16.5 units) than the normal range (4-10).

After collecting a blood sample, DNA was extracted by a manual method. The gene panel included the following genes: *MYH7, CHAT, CHRNA1, CHRNB1, CHRND, CHRNE, CHRNG, DPAGT1, GFPT1, LAMB2, MUSK, MYH3, MYH8, RAPSN, SCN4A, TNNI2, TNNT3, COLQ, DOK7, AGRN, MYBPC1, TPM2, PLEC, SLC35A3, ECEL1, GLE1, VIPAS39, VPS33B, PIEZO2, UBA1, CHST14,*and* FBN2.*Generally, targeted gene sequencing involves targeted capture and sequencing of protein-coding regions of genomes/genes. Mutations that are identified in the exonicregions are generally considered to be more pathogenic thanvariations that occur in non-coding regions. 

The libraries were sequenced to a meancoverage of >80-100X on anIllumina sequencing platform. The obtained sequences were aligned to the human reference genome (GRCh37/hg19) in the BWA software package (10, 11).They were also analyzed using the Picard and GATK-Lite toolkit(12, 13) to identify variants in targeted genes,associated with clinical presentations. Clinically relevant mutations were annotated, using the variants published in the literature, as well as a set of variant databases, includingClinVar (http://www.ncbi.nlm.nih.gov/clinvar/), OMIM, GWAS, HGMD, and SwissVar (http://swissvar.expasy.org/). Only non-synonymous and splice-site variants, found in the arthrogryposisand CMS panel genes, were used for clinical interpretations. 

The results showed a homozygous single base pair deletion at exon 12 of the *CHRNE* gene (chr17:4802186delC),causing the frameshift and premature truncation of 64 amino acid proteinsdownstream of codon443 (p.Glu443LysfsTer64; ENST00000293780). No other variantswere detectedthat need to be reported.

## Discussion

The muscle AChRcontainsfive subunits of four different types (twoalpha,one beta, onegamma, and onedelta). Mutations inthe AChR ε-subunit are more frequent than mutations of alpha, beta, and delta subunits. Mutations in these subunits are frequentlyrelated to a severe phenotype. The*CHRND*gene mutations should be considered in severe and early cases of the disease. They clinically resemble rapsyn phenotypes with recurrent episodic apneas (16).In this regard, Hoffmann et al. and Morgan et al. showed that mutations in the *CHRNG* gene resulted in Escobar syndrome (EVMPS; 265000), as well aslethal multiple pterygium syndrome (LMPS; 253290). 

The *CHRNG* gene encodes the AChRgamma subunit and is expressed before week 33 of pregnancy in humans, but is replaced by theε-subunit (100725) in the perinatal period. Therefore, the gamma subunit not only contributes to neuromuscular signal transduction, but is also important for neuromuscular organogenesis. Mutations in the*CHRNA *gene may cause disorders, such as LMPSand CMS (slow- and fast-channel CMS).Also, mutations in the*CHRNB *gene may lead to slow-channel CMS. Homozygous or compound heterozygous mutations in the *CHRNE* gene (OMIM entry #100725) have been implicated in CMS4C, associated with AChRdeficiency and fast-channel CMS4B (OMIM entry #616324). This *CHRNE* variant has been previously identified in five CMS patients with AChRdeficiency in Southeast of Iran (Khuzestan)(14, 15). It has not been submitted to the 1000 Genome Project database and is conserved across mammals.

Based on the abovementioned findings, this*CHRNE*variant can be classified as a pathogenically significant variant that may be included ingeneticscreenings. Sequencing of this variant in theparents(an *in vitro*strategy) and other affected and non-affected members of the family is recommended to confirm its significance.It seems that mutations in*CHRNE*genes are common in the Iranian population,which may contribute to thepathogenicity of CMS. Such variants may be detected via further targeted and wholegenome sequencing. 

**Table 1:** Conjenital myasthenic syndrome (CMS)

Genes	Sites	Percentage of pathogenic variants	Products	Disorders
*CHRNE*	17p13.2 at the short (p) arm of chromosome 17 at position 13.2	50%	AChR epsilon subunit	1- Multiple pterygium syndrome lethal type (MUPSL) 2- Slow-channel congenital myasthenicsyndrome (SCCMS) 3- Fast-channel congenital myasthenic syndrome (FCCMS)
*CHRND*	2q37.1 at the long (q) arm of chromosome 2 at position 37.1	1%		1- MUPSL 2- SCCMS 3- FCCMS
*CHAT*	10q11.23 at the long (q) arm of chromosome 10 at position 11.23	4-5%	Choline acetyltransferase	1- CMS
*MUSK*	9q31.3 at the long (q) arm of chromosome 9 at position 31.3	1%	Muscle-specific tyrosine kinase receptor	1- CMS
*ALG14*	1p21.3 at the short (p) arm of chromosome 1 at position 21.3	1%	Subunits of UDP-GlcNActransferase	1- CMS
*CACNB2 *	10p12.33-p12.31 at the short (p) arm of chromosome 10 between positions 12.33 and 12.31	1%	Voltage-gated calcium channel superfamily	1-Lambert-Eaton myasthenic syndrome 2- Brugada syndrome
*SYT2*	1q32.1 at the long (q) arm of chromosome 1 at position 32.1		Calcium sensor 1	1-CMS 2- Presynaptic CMS with or without motor neuropathy
*COLQ*	3p25.1 at the short (p) arm of chromosome 3 at position 25.1	10-15%	Acetylcholinesterase	
*DOK7*	4p16.3 at the short (p) arm of chromosome 4 at position 16.3	10-15%	Connection between nerve cells and muscle cells	CMS
*RAPSN*	11p11.2 at the short (p) arm of chromosome 11 at position 11.2	20%	AChR	CMS
*CHRNB1*	17p13.1 at the short (p) arm of chromosome 17 at position 13.1	1%	AChRbeta subunit	SCCMS
*AGRN *	1p36.33 at the short (p) arm of chromosome 1 at position 36.33	1%	1- LamininG 2- Kazal-type serine protease inhibitor 3- Epidermal growth factor domains	CMS
*SCN4A*	17q23.3 at the long (q) arm of chromosome 17 at position 23.3	1%		Sodium channel alpha subunit
*B3GLCT*	13q12.3 at the long (q) arm of chromosome 13 at position 12.3	10-15%	Beta 3-glucosyltransferase (B3Glc-T)	CMS

**Table 2:** *Arthrogryposis multiplex congenita*

Genes	Sites	Products	Disorders
*ECEL1*	2q37.1 at the long (q) arm of chromosome 2 at position 37.1	Endopeptidases (M13) and zinc-containing type II integral-membrane proteins	1- Arthrogryposis multiplex congenita 2- Autosomal recessive distal arthrogryposis (type 5D)
*TPM2*	9p13.3 at the short (p) arm of chromosome 9 at position 13.3	β-tropomyosin (part of tropomyosin)	1- Cap myopathy 2- Congenital fiber-type disproportion 3- Distal arthrogryposis type 1 4-Nemalinemyopathy 5-Sheldon-Hall syndrome 6- Arthrogryposis multiplex congenita
*MYBPC1*	12q23.2 at the long (q) arm of chromosome 12 at position 23.2	Myosin-binding protein C (a slow skeletal muscle isoform)	1- Arthrogryposis multiplex congenita
*ADCY6*	12q13.12 at the long (q) arm of chromosome 12 at position 13.12	Adenylyl cyclase	1- Arthrogryposis multiplex congenita
*KRT17*	17q21.2 at the long (q) arm of chromosome 17 at position 21.2	Keratin 17 (K17)	1- Arthrogryposis multiplex congenita
*KRT16*	17q21.2 at the long (q) arm of chromosome 17 at position 21.2	Keratin 16 (K16)	1- Arthrogryposis multiplex congenita
*RBPJ*	4p15.2 at the short (p) arm of chromosome 4 at position 15.2	RBP-J	1- Arthrogryposis multiplex congenita
*VIPAS39*	14q24.3 at the long (q) arm of chromosome 14 at position 24.3	Sorting of lysosomal proteins	1- Arthrogryposis multiplex congenita 2- Renal dysfunction 3- Cholestasis type 2
*NR0B1*	Xp21.2 at the short (p) arm of the X chromosome at position 21.2	DAX1	1- Arthrogryposis multiplex congenita
*TINF2*	14q12 at the long (q) arm of chromosome 14 at position 12	Telomere protein protectors	1- Arthrogryposis multiplex congenita
*CLCN1*	7q34 at the long (q) arm of chromosome 7 at position 34	Chloride channels	1- Arthrogryposis multiplex congenita
*RBPJ *	4p15.2 at the short (p) arm of chromosome 4 at position 15.2	RBP-J (part of the Notch signaling pathway)	1- Arthrogryposis multiplex congenita
*ARHGAP31*	3q13.32-q13.33 at the long (q) arm of chromosome 3 between positions 13.32 and 13.33	GTPase-activating protein (GAP)	1- Arthrogryposis multiplex congenita

## In Conclussion

Based on the above mentioned findings, this CHRNE variantcan be classified as a pathogenically significant variant that maybe included ingeneticscreenings. Sequencing of this variant in theparents(an in vitrostrategy) and other affected and non-affected members of the family is recommended to confirm its significance.It seems that mutations inCHRNEgenes are common in the Iranian population,which may contribute to thepathogenicity of CMS. Such variants maybe detected via further targeted and wholegenome sequencing. 
